# Iguratimod efficacy in palindromic rheumatism treatment

**DOI:** 10.1002/iid3.932

**Published:** 2023-06-28

**Authors:** Fangfang Yuan, Junhong He, Jing Luo, Xin Zhang, Jixia Lin, Yahui Chen, Haiyan Huang, Qiong Yang

**Affiliations:** ^1^ Department of Rheumatism and Immunology Ningbo No. 6 Hospital Ningbo China; ^2^ Department of Pharmacy Ningbo NO.6 Hospital Ningbo China

**Keywords:** clinical symptoms, disease control, flare frequency, iguratimod, palindromic rheumatism, significant efficacy

## Abstract

**Background:**

As a new immunomodulator for rheumatoid arthritis, iguratimod (IGU) also has therapeutic potential in other immune diseases. In this study, we determined the effects of IGU on disease control in patients with palindromic rheumatism (PR).

**Methods:**

Patients with PR were divided into Control group (Ctrl group) and an IGU treatment (IGU group) groups. Drug efficacy was evaluated according to the frequency of PR attacks (monthly), the visual analog scale (VAS) score of patient pain, and clinical symptoms.

**Results:**

The drug positivity and disease control rates of the IGU group (100.00% and 90.91%, respectively) were significantly higher than those of the Ctrl group (61.11% and 5.56%; *p* = .002 and *p* < .001, respectively). The median number of PR flares and the VAS score of patients in the Ctrl group decreased from 3.00 (1.00–15.00) to 0.83 (0.00–12.00) and from 5 (4–6) to 4 (1–6), respectively. In the IGU group, the median number of PR attacks decreased from 4.50 (2.00–15.00) to 0.00 (0.00–0.33), and the VAS score decreased from 5 (4–6) to 0 (0–2). The IGU group exhibited a significant reduction in PR flare frequency and improvement in the VAS value (*p* < .001 and *p* < .001, respectively).

**Conclusion:**

Our study is the first to describe the efficacy of IGU in PR treatment. IGU can significantly reduce the number of PR flares and improve the clinical symptoms of patients with PR.

## Graphical abstract

We analyzed the potential of iguratimod (IGU) as a therapy for palindromic rheumatism (PR), the treatment of which has until now remained controversial. Forty patients with PR were divided into control and IGU groups, and drug efficacy was determined by PR flare frequency and patient VAS scores. IGU significantly improved the incidence rate and clinical symptoms of PR. IGU is a promising new candidate for PR treatment.

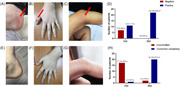

## 1 INTRODUCTION

Palindromic rheumatism (PR) is an immune disease characterized by the recurrent and self‐resolving inflammation of joints and tissues around the joints. The sites of PR onset are migratory and typically involve single joints, such as the knee, wrist, metacarpophalangeal, and proximal interphalangeal joints. PR flares are unpredictable and can occur several or even dozens of times per month. Joint pain, redness, swelling, and limited movement might last for several days during a PR flare‐up, causing both physical and mental pain.^1^, ^2^ PR and rheumatoid arthritis (RA) are closely related, but they are different diseases. Almost 50% of patients with PR will eventually progress to early RA.^3^, ^4^, ^5^ Similar rates of anti‐cyclic citrullinated peptide (anti‐CCP) antibody positivity have been reported in PR and RA. In addition, patients with PR exhibit an increased prevalence of HLA‐DR shared epitope alleles, which are more likely to cause RA.^6^, ^7^ Therefore, some drugs for RA can be used to treat PR. Currently, drugs used to treat PR are mainly divided into the following categories: nonsteroidal anti‐inflammatory drugs (NSAIDs), disease‐modifying anti‐rheumatic drugs (DMADs), antimalarials (hydroxychloroquine), and biological agents. However, the optimal treatment regimen for patients with PR remains controversial.

Iguratimod (IGU) is a novel small molecule compound that regulates the immune system. It inhibits the production of various inflammatory factors and immunoglobulins without affecting B cell number and proliferation.^8^, ^9^ Several clinical trials have verified its effectiveness and safety in RA treatment, suggesting great therapeutic potential for other immune diseases.^10^, ^11^ Therefore, in this study, we review the clinical data of patients with PR from 2020 to 2022 and verify the role of IGU in the treatment of refractory PR by comparing its therapeutic effects with those of traditional drugs. The aim of this study was to provide new options and clinical support for PR treatment.

## 2 METHODS

### 2.1 Patients

This retrospective study was conducted at the Department of Rheumatism and Immunology, Ningbo No. 6 Hospital, Ningbo, China. All patients with PR from 2020 to 2022 were included. Two senior rheumatologists repeatedly confirmed the PR diagnosis for patients included in the study according to patient clinical manifestations and multiple clinical encounters. None of the patients in the study took other drugs during the 3 months before the treatment regimen. All treatment regimens were disclosed to the patients, whose wishes were followed as much as possible to ensure drug safety. Patients in the control (Ctrl) group were treated with traditional drugs, including hydroxychloroquine or NSAIDs. Patients in the IGU group believed that traditional drugs were ineffective and chose IGU for treatment. The study and use of IGU in PR were approved by the Ethics Committee of the Ningbo Sixth Hospital. Informed consent for this study was irrelevant, as it was a retrospective study.

### 2.2 PR diagnostic criteria

As there is currently no recognized diagnostic standard for PR, the diagnosis provided by Guerne in 1992^11^ was used to define the inclusion/exclusion criteria for patients in this study:

a) Recurrent and unpredictable monoarthritis or oligoarthritis attacks or inflammation of the periarticular tissue, with a course of more than 6 months.
b) At least one PR flare confirmed by the physician.
c) More than three joints involved in previous PR attacks.
d) Other types of arthritis were excluded.

### 2.3 Treatment regimen

Patients in the Ctrl group were treated with hydroxychloroquine or meloxicam. The hydroxychloroquine dosing regimen was 0.1 g twice daily, lasting for 3 months. The meloxicam dosing regimen was 7.5 mg daily, which was changed to 7.5 mg every other day after 1 month. Given the lack of reports on PR treatment using IGU, after discussion by experts in the rheumatology department, the treatment regimen for patients in the IGU group was set to 25 mg twice daily for 14 days, which was subsequently changed to 25 mg daily. The IGU dosage was immediately adjusted in cases of adverse reactions.

### 2.4 Clinical data

The clinical information of patients with PR from 2020 to 2022 was collected through the hospital information system. We then analyzed their disease activity 3 months after taking the medication. Drug efficacy was evaluated based on the frequency of PR attacks (monthly), the visual analog scale (VAS) score of pain, and clinical symptoms. A reduction in the frequency of PR flares indicated a positive drug outcome; otherwise, the outcome was negative. The disease was considered controlled if no PR attack occurred within 3 months. Changes in PR flare frequency and VAS values showed the efficacy of the drugs. The laboratory test data of all patients were also collected and analyzed.

### 2.5 Statistical analysis

Demographic variables are expressed as means ± standard deviation (SD). The frequency of PR flares and the VAS values are expressed as median values (min–max). The drug positivity rate and disease control rate of the two groups were analyzed using Fisher's exact test. Changes in flare frequency and VAS values in the two groups were analyzed using the Kolmogorov–Smirnov test. All statistical analyses were performed using GraphPad Prism 9. Statistical significance was set at *p* ≤ .05.

## 3 RESULTS

Between 2020 and 2022, 49 patients with PR were admitted to our hospital. Among them, 41 patients were willing to receive oral drug treatment; however, one patient was excluded from the study after IGU treatment caused persistent abnormal liver function. Of the 40 patients in this study, 18 (45.00%) were enrolled in the Ctrl group, and 22 (55.00%) were in the IGU group. The two groups exhibited no significant differences in demographic characteristics, disease activity, or laboratory test results. The knee joint was the predominant location of the first PR flare, followed by the wrist and interphalangeal joints (Table 1). In addition, the rheumatoid factor and anti‐CCP antibody tests were negative in all patients with PR.

**Table 1:** Descriptive data of the clinical and demographic variables of patients with PR before treatment.

	Ctrl (*n* = 18)		IGU (*n* = 22)	
Item	Mean	SD	Mean	SD
Age	32.72	9.615	37.64	13.44
Course of disease (months)	50.50	49.44	44.46	40.85
Number of joints involved	7.00	2.54	8.23	2.64
Laboratory test				
WBC	6.47	2.09	6.19	1.98
HB	139.70	15.93	138.00	16.17
PLT	251.30	48.45	235.10	51.15
CRP	5.67	5.31	9.56	16.39
ESR	20.61	11.71	20.36	15.74
ALT	22.56	19.73	22.18	6.46
AST	16.56	5.14	19.64	5.31

	**Median**	**(Min–max)**	**Median**	**(Min–max)**
Frequency of flares	3.0	(1.0–15.0)	4.5	(2.0–15.0)
VAS	5.0	(4.0–6.0)	5.0	(4.0–6.0)

	***n***	**%**	***n***	**%**
Gender				
Female	8	44.44	8	36.36
Male	10	55.56	14	63.64
First joint to flare				
Knee	8	44.44	9	40.91
Wrist	6	33.33	3	13.64
Interphalangeal joints	2	11.11	5	22.73
Toe	1	5.56	2	9.09
Hip	1	5.56	2	9.09
Elbow	0	0.00	1	4.55

Abbreviations: ALT, alanine aminotransferase; AST, aspartate transaminase; CRP, C–reactive protein; ESR, erythrocyte sedimentation rate; HB, hemoglobin; PLT, blood platelets; PR, palindromic rheumatism; VAS, visual analog scale; WBC, white blood cell.

**Figure 1:**
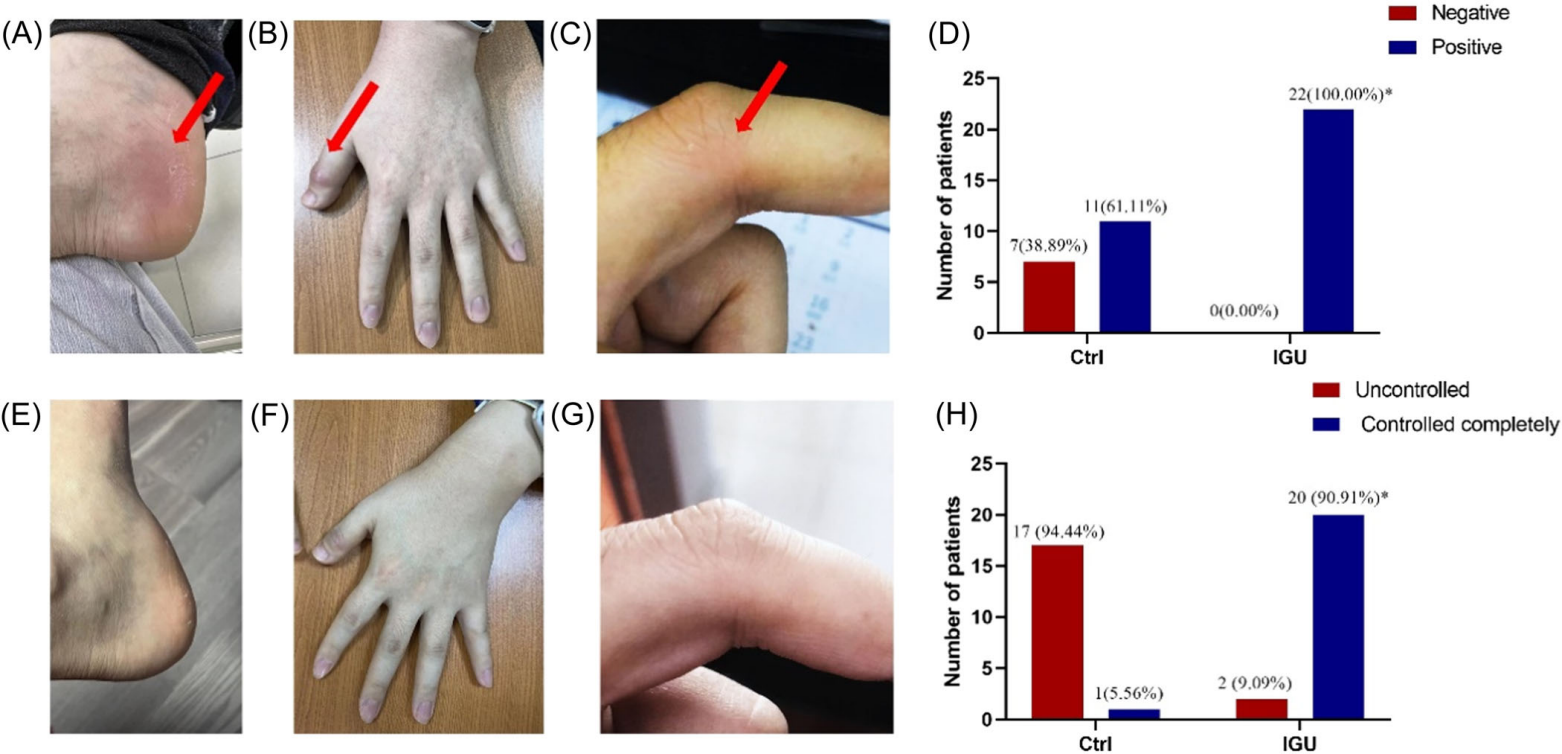
(A–C) Clinical manifestations of patients with PR, including redness, swelling, and pain in single joints or periarticular tissues. (D) The number and percentage of patients with drug positivity in the Ctrl and IGU groups. (E–G) The joint symptoms resolved and did not reappear after 3 months of treatment. (H) The number and percentage of patients with complete disease control in the Ctrl and IGU groups. PR, palindromic rheumatism.

After 3 months of treatment, the number of patients with drug positivity and complete disease control in the Ctrl group was 11 (61.11%) and 1 (5.56%), respectively. Conversely, the number of patients with drug positivity and complete disease control in the IGU group was 22 (100.00%) and 20 (90.91%), respectively. Therefore, the drug positivity and disease control rates were significantly higher in the IGU group than those in the Ctrl group (*p* = .002 and *p* < .001, respectively) (Figure  1D,H). The main clinical manifestations in patients with PR were redness, swelling, and pain in a single joint or periarticular tissues (Figure  1A–C). After complete control of the disease, the joint symptoms resolved and did not reappear for 3 months (Figure  1E–G).

**Figure 2:**
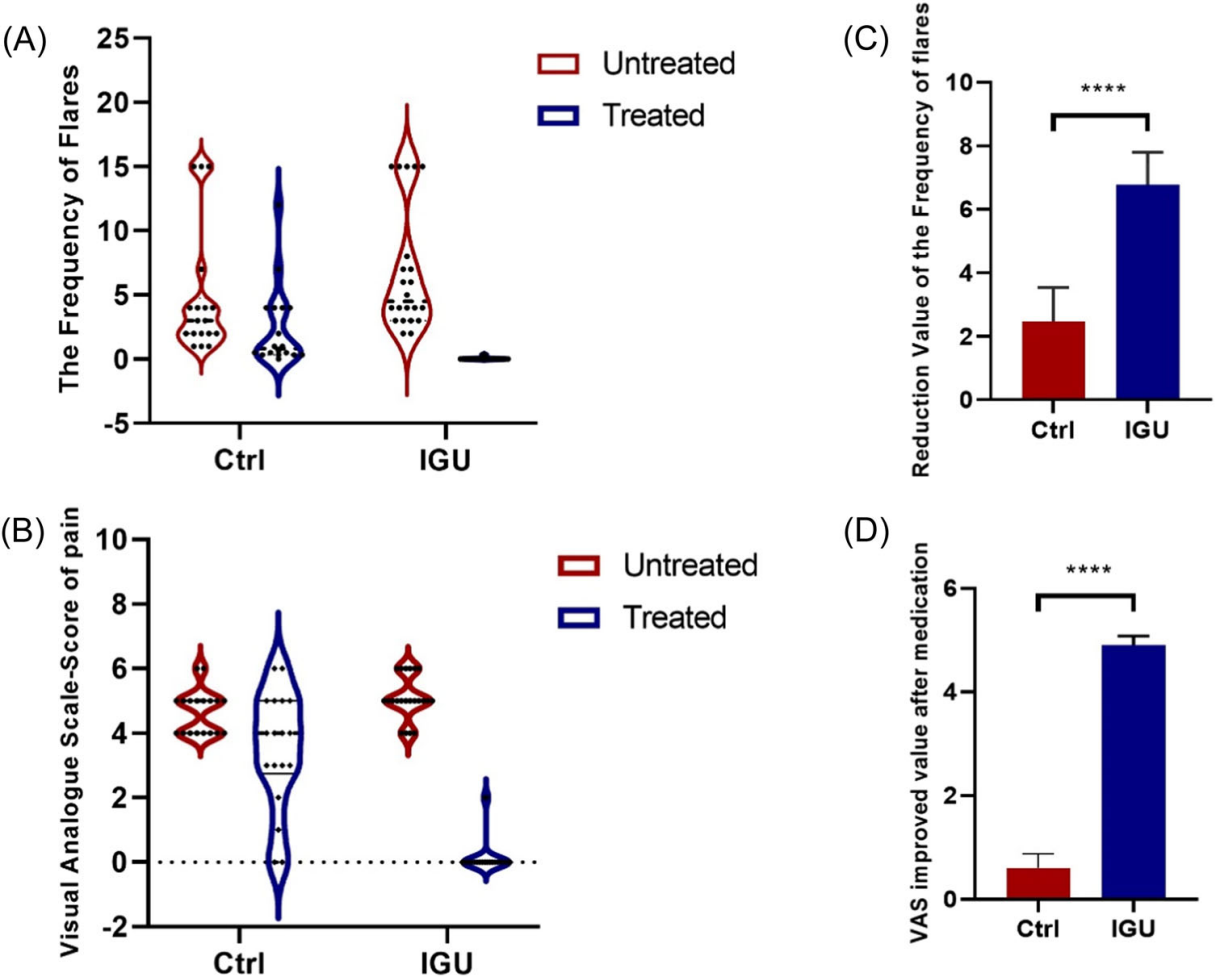
(A, B) The changes in frequency and VAS distribution of PR flares after medication in the Ctrl and the IGU group. (C, D) Reduction in PR flare frequency and VAS improvement after medication in the Ctrl and IGU groups. VAS, visual analog scale. ****p < .001.

In terms of curative effect, the median number of PR flares and the VAS score of patients in the Ctrl group decreased from 3.00 (1.00–15.00) to 0.83 (0.00–12.00) and from 5 (4–6) to 4 (1–6) after 3 months, respectively. In the IGU group, the median number of PR attacks decreased from 4.50 (2.00–15.00) to 0.00 (0.00–0.33), and the VAS value of patients decreased from 5 (4–6) to 0 (0–2), respectively. Compared with the Ctrl group, the PR flare frequency reduction (*p* < .001) and VAS score (*p* < .001) improved significantly in the IGU group (Figure  2).

## 4 DISCUSSION

Until recently, PR was considered a precursor symptom of early RA. However, the subsequent discovery of different inflammatory patterns, unique genetic predisposing factors, and imaging features of PR suggests that this disease cannot be simply classified as a stage of RA.^12^ Nevertheless, researchers have attempted to treat PR with anti‐RA drugs, such as NSAIDs, DMADs, glucocorticoids, and antimalarials, with some promising effects. Notably, the antimalarial drug hydroxychloroquine sulfate (HCQ) has gradually become a treatment option for long‐term management in patients with PR.^13^ A prospective study showed that 47.78% of patients with PR treated with HCQ achieved complete disease control.^14^ Another retrospective study confirmed that HCQ could effectively reduce the frequency and duration of PR flares.^15^ However, HCQ does not affect all patients with PR. Rituximab (CD20 monoclonal antibody, RXT) is a biological agent used to treat PR. A study in India found that RXT significantly improves PR in patients that previously exhibited a poor response to DMADs; however, some patients required multiple injections.^16^ Methotrexate has been the cornerstone of treatment for RA since 1980s. In 2021, Padhan and Thakur^17^ found that methotrexate addition effectively reduced the number and duration of PR episodes in patients with PR who were unresponsive to HCQ. Methotrexate could still play a role in PR similar to that in RA. Another retrospective study showed that methotrexate combined with DMARDs could control symptoms in patients with PR who were unresponsive to HCQ.^18^

IGU is a novel oral immunomodulator that can inhibit the production of a variety of inflammatory cytokines, including interleukin (IL)‐1, IL‐6, IL‐8, nuclear factor‐κB, and tumor necrosis factor (TNF).^8^, ^19^, ^20^, ^21^, ^22^ The results of Phase II and III clinical trials have confirmed the efficacy and safety of IGU in RA treatment. Compared with RXT, IGU is safer and does not require the exclusion of hepatitis, tuberculosis, infection, or other diseases before use. In addition, the properties of oral preparations make it easier to adjust the course of IGU treatment. More importantly, clinical studies have shown that IGU can cooperate with methotrexate to reduce inflammation, promote bone formation, and antagonize bone resorption in patients with RA.^23^ Compared with IGU alone, combination therapy with methotrexate is a more effective treatment for RA.^24^ IGU combined with methotrexate significantly inhibited the high expression of *RANKL* mRNA and inhibited the production of inflammatory factors, especially TNF‐α.^25^, ^26^ The synergistic effects of methotrexate and IGU suggest the potential of IGU for the treatment of recurrent rheumatic disease, similar to methotrexate. Before our study, no literature reports showed the use of IGU for PR treatment. Surprisingly, this study showed that IGU alone demonstrated a 100% reduction in PR frequency. The proportion of patients with complete disease control also exceeded 90%, which far exceeds the curative effect of HCQ. A significant improvement in the frequency of PR flares and VAS score was also observed in the IGU group. Notably, some patients (seven cases, 31.82%) in the IGU group had taken HCQ or NSAIDs in the past without any improvement. This indicates that IGU could still produce a therapeutic effect in refractory PR. It should also be noted that one patient was not included in the study. This patient withdrew from IGU treatment because of abnormal liver function and leukocyte count, but had no PR flares while taking IGU; instead, PR flares occurred 1 week after withdrawal. This phenomenon also confirms the role of IGU in PR treatment. In addition, IGU did not affect viral activity in two patients with hepatitis B virus.

PR is rare, and its diagnostic criteria are unclear, so it was difficult to include more patients with PR in this study; therefore, our results require further verification. Specifically, the strong therapeutic effect of IGU in PR treatment requires further clinical studies and a longer follow‐up period. More importantly, because of the lack of an accurate disease activity detection index for PR, PR flare frequency and VAS scores were used as the evaluation basis in this study. Furthermore, despite establishing the therapeutic effect of IGU in this study, we did not discuss the optimal therapeutic dose of IGU for PR or withdrawal of the drug. This will be the subject of future research. In addition, liver function and hematological adverse reactions are important factors that may limit the use of IGU, which should be monitored during clinical application.

## 5 CONCLUSION

IGU can significantly improve PR incidence rate and clinical symptoms, and it is also effective for refractory PR, where traditional drugs show weak effects. The optimal treatment regimen for IGU in PR requires further confirmation. Moreover, it is important to monitor liver function and the circulatory system during the clinical use of IGU.

## AUTHOR CONTRIBUTIONS

**Fangfang Yuan**: Data curation; investigation; project administration; resources; writing—original draft. **Junhong He**: Investigation; methodology; writing—original draft. **Jing Luo**: Project administration; resources; supervision; writing—review and editing. **Xin Zhang**: Software; visualization; writing—original draft. **Jixia Lin**: Formal analysis; resources. **Yahui Chen**: Data curation; formal analysis. **Haiyan Huang**: Investigation; methodology. **Qiong Yang**: Investigation; project administration; resources; supervision; writing—review and editing.

## CONFLICT OF INTEREST STATEMENT

The authors declare no conflict of interest.

## ETHICS STATEMENT

The study and use of iguratimod in palindromic rheumatism were approved by the Ethics Committee of the Ningbo Sixth Hospital. Informed consent for this study was not relevant, as it was a retrospective study.

## Data Availability

The original contributions presented in the study are included in the article. Further inquiries can be directed to the corresponding author.

## References

[1] Guerne PA , Weisman MH . Palindromic rheumatism: part of or apart from the spectrum of rheumatoid arthritis. Am J Med. 1992;93(4):451‐460. 1341421 10.1016/0002-9343(92)90177-d

[2] Mankia K , Emery P . What can palindromic rheumatism tell us? Best Pract Res Clin Rheumatol. 2017;31(1):90‐98. 29221602 10.1016/j.berh.2017.09.014

[3] Koskinen E , Hannonen P , Sokka T . Palindromic rheumatism: long term outcomes of 60 patients diagnosed in 1967‐84. J Rheumatol. 2009;36(9):1873‐1875. 19648311 10.3899/jrheum.090025

[4] Wajed MA , Brown DL , Currey HL . Palindromic rheumatism. clinical and serum complement study. Ann Rheum Dis. 1977;36(1):56‐61. 843112 10.1136/ard.36.1.56 PMC1006630

[5] Hannonen P , Müttönen T , Oka M . Palindromic rheumatism. A clinical survey of sixty patients. Scand J Rheumatol. 1987;16(6):413‐420. 3423751 10.3109/03009748709165412

[6] Salvador G . Prevalence and clinical significance of anti‐cyclic citrullinated peptide and antikeratin antibodies in palindromic rheumatism. an abortive form of rheumatoid arthritis? Rheumatology. 2003;42(8):972‐975. 12730510 10.1093/rheumatology/keg268

[7] Maksymowych WP , Suarez‐Almazor ME , Buenviaje H , et al. HLA and cytokine gene polymorphisms in relation to occurrence of palindromic rheumatism and its progression to rheumatoid arthritis. J Rheumatol. 2002;29(11):2319‐2326. 12415587

[8] Du F , Lü LJ , Fu Q , et al. T‐614, a novel immunomodulator, attenuates joint inflammation and articular damage in collagen‐induced arthritis. Arthritis Res Ther. 2008;10(6):136. 10.1186/ar2554 PMC2656239 19019215

[9] Tanaka K . Inhibitory effects of an anti‐rheumatic agent T‐614 on immunoglobulin production by cultured B cells and rheumatoid synovial tissues engrafted into SCID mice. Rheumatology. 2003;42(11):1365‐1371. 12810927 10.1093/rheumatology/keg381

[10] Lü L , Teng J , Bao C , et al. Safety and efficacy of T‐614 in the treatment of patients with active rheumatoid arthritis: a double blind, randomized, placebo‐controlled and multicenter trial. Chin Med J. 2008;121(7):615‐619. 18466681

[11] Hara M , Abe T , Sugawara S , et al. Efficacy and safety of iguratimod compared with placebo and salazosulfapyridine in active rheumatoid arthritis: a controlled, multicenter, double‐blind, parallel‐group study. Mod Rheumatol. 2007;17(1):1‐9. 17278015 10.1007/s10165-006-0542-y

[12] Mankia K , Emery P . Palindromic rheumatism as part of the rheumatoid arthritis continuum. Nat Rev Rheumatol. 2019;15(11):687‐95. 31595059 10.1038/s41584-019-0308-5

[13] Kavandi H , Hashemi SZ , Khalesi E , Khabbazi A . Treatment of palindromic rheumatism: a systematic review. Int J Clin Pract. 2021;75(11):e14868. 34525234 10.1111/ijcp.14868

[14] Emad Y , Anbar A , Abo‐Elyoun I , et al. In palindromic rheumatism, hand joint involvement and positive anti‐CCP antibodies predict RA development after 1 year of follow‐up. Clin Rheumatol. 2014;33(6):791‐797. 24623460 10.1007/s10067-014-2569-3

[15] Katz SJ , Russell AS . Palindromic rheumatism: a pre‐rheumatoid arthritis state? J Rheumatol. 2012;39(10):1912‐1913. 23028026 10.3899/jrheum.120995

[16] Raghavan P , Sreenath S , Cherian S , Shenoy PD . Efficacy of rituximab in resistant palindromic rheumatism: first report in literature. Clin Rheumatol. 2019;38(9):2399‐402. 31076945 10.1007/s10067-019-04578-2

[17] Padhan P , Thakur B . Effect of low dose methotrexate as an add‐on therapy in patients with palindromic rheumatism unresponsive to hydroxychloroquine: an observational study. Eur J Rheumatol. 2021;8(3):130‐132. 34101572 10.5152/eurjrheum.2021.20062 PMC9770407

[18] Khabbazi A , Mirza‐Aghazadeh‐Attari M , Goli MT , Mahdavi AM , Hajialilo M , Rashtchizadeh N . Is palindromic rheumatism a pre‐rheumatoid arthritis condition? low incidence of rheumatoid arthritis in palindromic rheumatism patients treated with tight control strategy. Reumatol Clín. 2021;17(1):7‐11. 30987884 10.1016/j.reuma.2019.01.002

[19] Kohno M , Aikawa Y , Tsubouchi Y , et al. Inhibitory effect of T‐614 on tumor necrosis factor‐alpha induced cytokine production and nuclear factor‐kappaB activation in cultured human synovial cells. J Rheumatol. 2001;28(12):2591‐2596. 11764202

[20] Xu Y , Zhu Q , Song J , et al. Regulatory effect of iguratimod on the balance of th subsets and inhibition of inflammatory cytokines in patients with rheumatoid arthritis. Mediat Inflamm. 2015;2015:1‐13. 10.1155/2015/356040 PMC4680115 26713003

[21] Yoon HY , Lee EG , Lee H , et al. Kaempferol inhibits IL‐1β‐induced proliferation of rheumatoid arthritis synovial fibroblasts and the production of COX‐2, PGE2 and MMPs. Int J Mol Med. 2013;32(4):971‐977. 23934131 10.3892/ijmm.2013.1468

[22] Tanaka K , Shimotori T , Makino S , et al. Pharmacological studies of the new antiinflammatory agent 3‐formylamino‐7‐methylsulfonylamino‐6‐phenoxy‐4H‐1‐benzopyran‐4‐o ne. 1st communication: antiinflammatory, analgesic and other related properties. Arzneimittel‐Forschung. 1992;42(7):935‐944. 1418059

[23] Yan XZ , Wang FL . Effects of iguramod combined with methotrexate on serum related cytokines and bone metabolism in patients with rheumatoid arthritis. J Changchun Univ Tradit Chin Med. 2018;34(2):369‐372.

[24] Wang X , Ma C , Li P , Zhao F , Bi L . Effects of iguratimod on the levels of circulating regulators of bone remodeling and bone remodeling markers in patients with rheumatoid arthritis. Clin Rheumatol. 2017;36(6):1369‐77. 28474138 10.1007/s10067-017-3668-8

[25] Wang WD , Sun YE . Preservation effect of meat product by natural antioxidant tea polyphenol. Cell Mol Biol. 2016;62(12):44‐50. 10.14715/cmb/2016.62.13.8 28040061

[26] Tan J , Dan J , Liu Y . Clinical efficacy of methotrexate combined with iguratimod on patients with rheumatoid arthritis and its influence on the expression levels of HOTAIR in serum. BioMed Res Int. 2021;2021:1‐7. 34805398 10.1155/2021/2486617 PMC8604587

